# Environmental risk associated with accumulation of toxic metalloids in soils of the Odra River floodplain—case study of the assessment based on total concentrations, fractionation and geochemical indices

**DOI:** 10.1007/s10653-023-01502-1

**Published:** 2023-02-23

**Authors:** Dorota Kawałko, Anna Karczewska, Karolina Lewińska

**Affiliations:** 1 Institute of Soil Science, Plant Nutrition and Environmental Protection, Wrocław University of Environmental and Life Sciences, Ul. Grunwaldzka 53, 50-357 Wrocław, Poland; 2 Department of Soil Science and Remote Sensing of Soils, Adam Mickiewicz University in Poznań, Ul. Krygowskiego 10, 61-680 Poznań, Poland

**Keywords:** Alluvial soils, Contamination, Igeo, Sequential extraction, BCR, Solubility, Reducible fraction

## Abstract

The floodplain soils are often heavily enriched in metal(loid)s released from the industrial areas. A related environmental risk depends on their total concentrations and the forms and conditions conducive to mobilization. This study was aimed to examine the concentrations of metal(loid)s in the Odra floodplain soils and to assess the risk associated with their possible contamination. In this study, topsoil and deeper soil layer samples were collected from the inter- and out-of-embankment zones. Total concentrations of Pb, Zn, Cu, As, Mn and Fe, and their extractable fractions were determined in 1 M NH_4_NO_3_ (actual solubility) and by BCR sequential extraction. The environmental risk was assessed based on total concentrations, according to legal regulations, geochemical enrichment indices and extractability of elements, with considering soil morphological features. Some topsoil samples from the inter-embankment zone turned out considerably enriched in Pb, Zn, Cu, and As, as confirmed by geochemical indices. Zn and As concentrations in some samples exceeded the permissible values defined by Polish law. Zn and Mn showed a high actual solubility, but a simple experiment proved that it can be efficiently reduced by liming. BCR fractionation showed that all the elements occurred mainly in reducible forms. Therefore, the risk of their release from the layers that do not indicate redoximorphic features was assessed as negligible. The study showed that such a complementary approach is needed to assess the real environmental risk in the case of soils considerably enriched in potentially toxic elements.

**Supplementary Information:**

The online version contains supplementary material available at 10.1007/s10653-023-01502-1.

## Introduction

Alluvial soils are often contaminated with heavy metals and metalloids. This applies in particular to the valleys of such rivers that flow through mining or industrial areas (Bednářová et al., 2015; Kanianska et al., 2022; Shaheen & Rinklebe, 2014). Several studies confirmed high concentrations of metal (loid)s in floodplain soils of the Odra River (the Oder), the second largest river of Poland (Ciszewski & Grygar, 2016; Jabłońska-Czapla et al., 2016). Such an enrichment resulted from the fact that the Odra, in its upper reaches, and its tributaries flow through the Upper Silesia, the largest mining and industrial region in the country. Several historical and contemporary Zn-Pb mines and associated processing facilities were indentified as the sources of Zn, Pb and other potentially toxic elements (PTEs) in the sediments and soils along the Odra River valley (Aleksander-Kwaterczak & Helios-Rybicka, 2009). Additionally, two large coal mining districts: the Ostrava in the Czech Republic and the Upper Silesian coal basin in Poland, situated in the upper part of the Odra Basin have contributed to the enrichment of river sediments in various contaminants, including heavy metals (Ciszewski & Turner, 2009).

A significant enrichment of soils with PTEs may pose a threat to people and the environment, due to their possible transport into the food chain and leaching to natural water (Alloway, 2013; Bhatti et al., 2018; Korfali & Karaki, 2018; Rinklebe et al., 2019; Shaheen et al., 2020). Several indices of contamination, based on total levels of PTEs in soils, were proposed by various authors to assess a related risk (Kowalska et al., 2018; Lewińska & Karczewska, 2019), the most common of which are enrichment factor EF (Barbieri, 2016), potential ecological risk index RI (Hakanson, 1980), and the index of geoaccumulation Igeo (Müller, 1981) (Table S1).

Although total concentrations of PTEs might serve as indicators for an introductory assessment of soil contamination, it is known that this is not the information from which direct conclusions about a real environmental hazard can be drawn. The risk depends not only on the total content of elements, but to a large extent on their actual and potential mobility and bioavailability (Alloway, 2013; Dradrach et al., 2020; Kabata-Pendias & Szteke, 2015; Kicińska, 2019). Speciation of PTEs in soils depends on the kind of element and its affinity to various soil components, as well as on soil composition and its changing properties, such as pH, redox conditions, or the presence of chelating components (Alloway, 2013; Caporale & Violante, 2016; Ponting et al., 2021). The actual solubility of various elements in soils can be determined based on the results of single extractions with neutral salt solutions that trigger the desorption processes of weakly bound element species, mainly cations or anions (Kumpiene et al., 2017; Pueyo et al., 2004; Rao et al., 2008). The improvement in a single extraction approach is the diffusive gradients in thin films (DGT). This method allows taking into account the dynamic changes in element concentrations in soil solution as a result of its uptake by plants and continuous resupply from the solid phase (Zhang & Davison, 2015). However, the DGT method is more expensive and time-consuming than a simple extraction, and requires special equipment. Therefore, batch extraction is still most often used for the assessment of actual solubility of PTEs. Extraction with 1 M NH_4_NO_3_ is of particular importance as it has an ISO standard status (ISO 19730: 2008), however, some authors believe that it gives underestimated results (Rocco et al., 2018), and in the case of Cu they sometimes are overstated (Karczewska et al., 2015). If soils are subject to strongly changing conditions, the assessment on risk cannot be based on temporary soil properties, and therefore potential solubility of toxic elements should be examined, i.e., the pools that may be released from the solid phase due to the changes of soil conditions need to be determined. The most important factors to be considered, that can trigger the release of elements, are acidification and the drop in redox potential. At low pH, cationic forms of metals will be desorbed (Alloway, 2013), and additionally, in a strongly acidic environment (pH below ca. 4.0, depending on soil properties), iron (hydroxy)oxides, which are the main sink of anionic forms of metalloids such as As, may partially dissolve, thus releasing anions into the soil solution (Wenzel, 2013). Redox potential is another important factor that can control the release of PTEs accumulated in the soil. The elements occluded in Mn and Fe (hydroxy)oxides, especially amorphous ones, will be released under reducing conditions (Frohne et al., 2014; Kelly et al., 2020; Lewińska et al., 2019; Ponting et al., 2021; Ratié et al., 2019; Xu et al., 2017). On the other hand, the oxidation processes in soils and sediments remaining initially under reducing conditions may lead to the transformation of insoluble sulfides into soluble sulfates of metals such as Cd, Zn or Cu (Du Laing et al., 2009; Tack, 2010). Another mechanism of the potential release of metal (loid)s is based on chelation, so that the chelating substances present in soil solution can bind and dissolve the elements, particularly those associated with soil organic matter.

The potential solubility of elements in soil or sediment can be assessed based on the fractionation analysis, which determines the amounts of elements bound to various soil components that can be released by various mechanisms. Sequential extraction is the most common approach that can be used to examine the operationally defined fractions of metal (loid)s in soils and to predict possible changes in their solubility associated with changing soil conditions. Dozens of different sequential extraction procedures have been developed so far (Rao et al., 2008; Zimmerman & Weindorf, 2010), of which the modified BCR scheme is considered one of the most reliable and precise (Rauret et al., 1999), and therefore it pretends to become an ISO standard. It distinguishes three potentially soluble fractions of elements: acid-soluble (*F*1), reducible (*F*2) and oxidizable (*F*3). The residue from the extraction of these three fractions are the forms considered to be insoluble, often referred to as a residual fraction (*F*4). The sum of *F*1 + *F*3 + *F*3 fractions makes a pool of element that can be potentially mobilized, therefore its share in the total concentration of the element in soil can be defined as a potential mobility factor (PMF) (Rinklebe and Shaheen, 2014; 2017; Shaheen & Rinklebe, 2014).

The knowledge on speciation of metals and metalloids in soils of river valleys is particularly important because these soils, periodically flooded, are highly susceptible to the changes in redox conditions and related changes in the solubility of elements (Ponting et al., 2021). Many authors analyzed the forms of various elements in contaminated alluvial soils, using different sequential extraction procedures (Aiyesanmi et al., 2020; Barać et al., 2016; Frentiu et al., 2009; Izquierdo et al., 2013). They found that the fractions of elements in freshly flooded soils differ from those in long-ago flooded ones (Barać et al., 2016; Frohne et al., 2014), and the proportion of readily soluble forms is usually the highest in the surface soil layers (Frentiu et al., 2009). The results of many studies carried out with poorly contaminated soils indicated a low potential mobility of most metals and a high share of the residual fraction (Barać et al., 2016; Li et al., 2015). The exceptions were usually: Pb with the reducible fraction often being dominant, and Zn and Cd found usually in considerable amounts in the acid-soluble and reducible fractions, in particular shortly after the flood (Barać et al., 2016). Similar observations regarding Pb and Cd were reported by Rinklebe and Shaheen (2014) and Shaheen and Rinklebe (2014) from the Central Elbe River valley, by Różanski (2013) from Fordonska valley, by Kanianska et al., (2022) from Štiavnica River floodplain, as well as from several other studies (Shaheen et al., 2015, 2020). However, different authors reported different results regarding Cu. This metal has a high affinity to humic substances and therefore it often occurs in soils in the forms associated with organic matter. Though Różanski (2013) and Rinklebe and Shaheen (2017) indicated that the shares of organically bound Cu forms were in Fluvisols low, and the reducible forms of Cu were the main ones.

This short review shows that the solubility and forms of potentially toxic metal(loid)s in alluvial soils may differ widely. Therefore, the aim of our research was to determine the actual solubility and fractionation patterns of elements, particularly those of mainly anthropogenic origin, i.e., Pb, Zn, Cu, As, as well as two mainly lithogenic metals, i.e., Fe and Mn, in the alluvial soils of the Odra River valley (SW Poland), and to discuss the risk of their mobilization from considerably enriched soils. The patterns of fractionation were compared between various soil horizons and localities, i.e., in the inter-embankment zone, periodically flooded, and those outside the embankments. The associated environmental risk was discussed, taking into account the conclusions based on the solubility and fractionation analysis and those drawn from the calculated indices of geochemical enrichment.

## Materials and methods

### Location of sampling sites

**Fig. 1:**
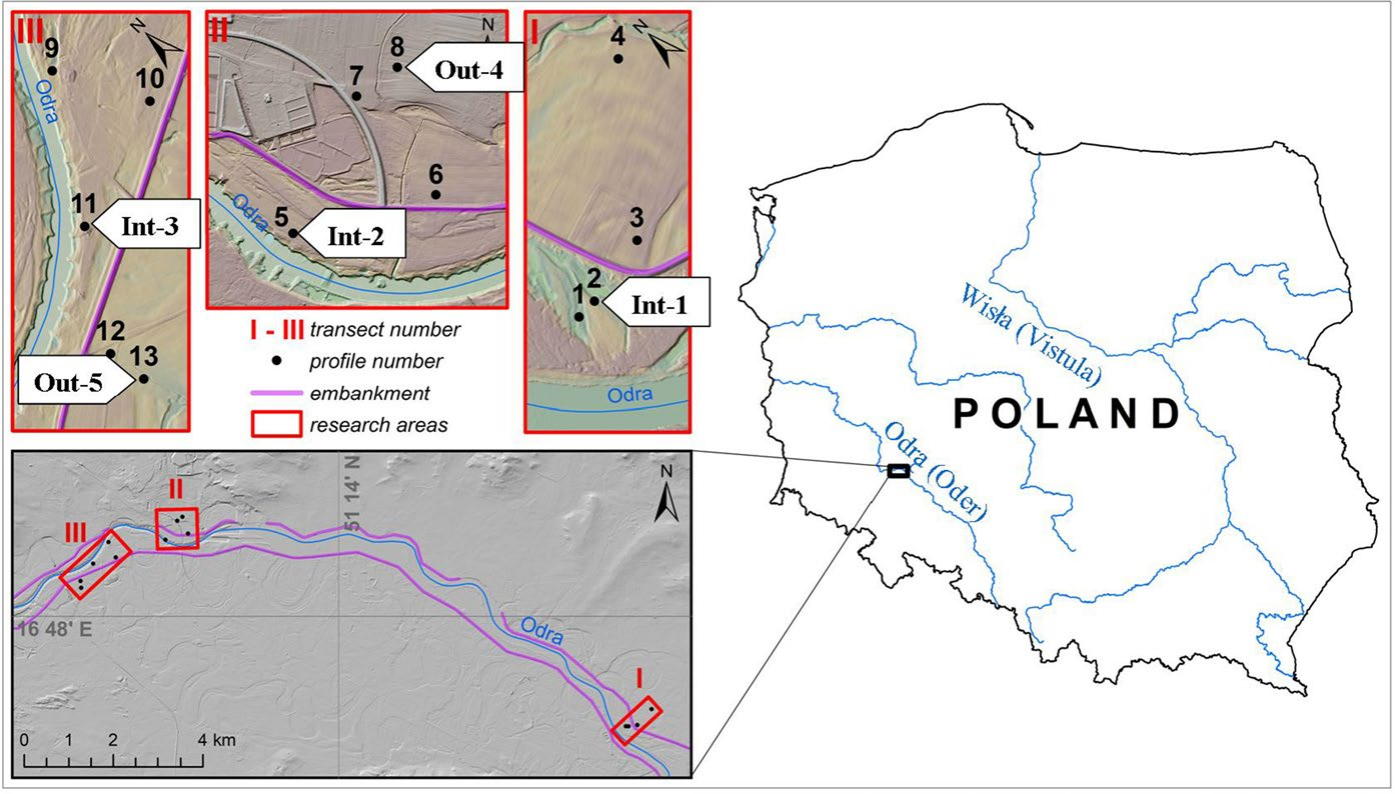
Location of soil sampling sites. Arabic numerals refer to the numbering within a larger set of soil profiles, which were studied in terms of basic soil properties and morphological features and presented in another paper

Soil samples representative of top soil layers and deeper horizons were collected from five profiles located in the Odra River valley, in its middle course, downstream of Wrocław (Fig. 1). All the profiles represented lands used for agriculture, i.e., pastures or arable lands. Three profiles (No. 1–3) were located in the inter-embankment zone (Int), in the areas used as grasslands (meadows and pastures), occasionally flooded, and two (No. 4 and 5)–out of the embankment (Out), in the lands used as plowed fields. Favorable conditions for agricultural use in those sites were created by channeling the river and construction of embankments at the beginning of the, twentieth century (Kabała et al., 2011; Kawałko et al., 2021). The main criterium for selecting the samples for analysis from a much larger set, described in another study by Kawałko and Karczewska (submitted), was the presence of considerably high concentrations of metal (loid)s, so that it was possible to perform the analyses with a satisfactory precision and accuracy. Therefore, the samples with a heavier texture were taken into account, while sandy samples, with a very low total content of the examined elements, were not analyzed.

### Soil sampling and analysis of basic soil properties

Soil pits were dug to a depth of 1.5 m or to the level of groundwater. Morphology of soil profiles was described according to World Reference Base for soil resources (WRB, 2022). Three of the soil were classified as Eutric Fluvic Cambisols, and two others—as Cambic Fluvic Phaeozem and Eutric Stagnosol (Table 1). Soil samples were collected from the distinguished horizons and transported to laboratory. For this study, focused on the solubility of metal(loid)s, two kinds of samples were chosen: those representative of the surface soil layers, with humus accumulation, and for comparison, the samples from deeper horizons, with either gleyic (r) or stagnic (g) features, from various depths, down to 170 cm (Table 1). The samples were prepared for analysis according to the rules applied in soil science (Tan, 2005). Visible roots and macrofauna were removed, soil samples were homogenized, air dried for several days, and ground to pass through a 2-mm sieve. Soil texture was determined by a combined sieve and hydrometer method (Papuga et al., 2018) and categorized according to USDA classification. Chemical soil properties were determined in the representative aliquots of samples, ground to a powder. Soil pH was measured in a suspension in 1 M KCl (1:2.5; v/v). Organic carbon (Corg) was determined by a dry combustion method (Vario MacroCube, Elementar). Cation exchange capacity (CEC) was determined as a sum of acidity and basic cations extracted with 1 M NH_4_OAc, pH 7.0 (Tan, 2005). Pseudototal concentrations of metal(loid)s: Pb, Zn, Cu, As, Mn and Fe, called further “total” were determined by ICP-AES (iCAP 7400, Thermo Fisher Scientific) after microwave digestion with aqua regia (ISO 11466: 1995). All analyses were made in triplicates. The accuracy of results was checked with reference materials: CRM 027 and CNS 392 (Sigma-Aldrich), certified for aqua-regia digestion. The recoveries of elements ranged 94–109% which we considered satisfactory.

**Table 1:** List of profiles and samples, their basic properties and total concentrations of the analyzed elements compared to European geochemical background

Profile	Land use/and soil type acc. to WRB (2022)	Horizon	Depth.cm	Clay %	Textural class^a)^	Corg.g/kg	pH	CEC cmol^+^/kg	Pb	Zn	Cu	As	Mn	Fe
									mg/kg					
Int-1	Meadow/Eutric Gleyic Fluvic Cambisol	Ah	0–10	24	SiL	29	3.7	21.0	35.5	138	19.6	13.6	707	24,900
		IIICr	58–75	21	SiL	6	4.0	14.7	13.8	94	13.4	10.4	1210	23,600
Int-2	Pasture/Eutric Stagnic Fluvic Cambisol	Ah	0–6	7	SL	66	5.3	11.4	34.5	324	28.1	10.6	413	12,600
		Ag	16–30	12	SiL	118	5.0	14.1	117	724	55.3	26.1	680	23,500
		Bwg	45–70	21	SiL	18	5.3	18.3	39.9	305	27.8	28.3	1310	26,100
Int-3	Meadow/Cambic Fluvic Phaeozem	Ah	0–15	25	SiL	116	4.6	18.2	162	914	74.7	38.2	826	29,200
		ABw	30–55	20	SiL	41	5.3	19.1	266	1150	59.2	45.8	1170	32,300
		IIICg	145–170	24	SiL	2.6	5.0	15.2	8.3	96	9.5	7.4	1100	26,700
Out-4	Arable land/Eutric Stagnosol	Ap	0–22	12	SL	18	6.1	10.4	12.4	80	5.0	13.9	892	47,500
		Cr1	80–105	27	L	2.4	7.4	15.1	9.5	105	9.4	2.5	41.5	16,900
Out-5	Arable land/Eutric Stagnic Fluvic Cambisol	Ap1	0–25	28	SiCL	21	3.4	18.9	20.1	110	17.6	12.7	916	26,800
		Cg1	75–110	56	C	13	3.8	34.3	16.8	110	18.5	14.1	2010	50,900
European geochemical background^b)^	Topsoil								15.0	48	12.0	6.0	382	19,600
	Subsoil								10.0	44	13.9	5.0	337	21,100

^a)^Textural classes according to USDA: SiL–silt loam, SL–sandy loam, L–loam, SiCL—silty clay loam, C–clay

^b)^European geochemical background for soils according to FOREGS (Salminen, 2004)

### Single and sequential extraction

A single extraction with 1 M NH_4_NO_3_ solution was applied to determine the actual solubility of elements in soils, using a soil to solution ratio of 1:2.5 (m/V) for 120 min at (20 ± 2) °C, under overhead shaking, according to ISO 19730. For determination of operationally defined fractions of metal (loid)s, the standardized, three-step BCR sequential extraction method (Rauret et al., 1999), was applied. Three fractions of elements were sequentially extracted: acid-soluble (*F*1), reducible (*F*2) and oxidizable (*F*3). More detailed information on extraction steps is provided in the Table S1 (Suppl. Materials). The residue after extraction, considered a residual fraction (*F*4), was digested with aqua regia, as described above. The concentrations of elements in all extracts and digests were determined by ICP-AES (iCAP 7400, Thermo Fisher Scientific). The analysis was considered properly done when the recovery (the sum of *F*1:*F*4 fractions related to total) was in the range of 95–105%. Finally, for each sample, the PMFs of all elements were calculated, defined as the share of potentially mobilizable fractions (*F*1 + *F*2 + *F*3) in total concentration in soil.

### Assessment of environmental risk

Two different approaches were used to assess the environmental risk. The first approach involved calculation of three geochemical contamination indices based on total concentrations of PTEs in soils, i.e., enrichment factor (EF), potential ecological risk index (RI) and the index of geoaccumulation (Igeo). Related formulas and the ranges of values corresponding with various risk categories are given in Table S2. The second approach, that emphasized the importance of soil pH for the assessment of a real environmental risk, involved a method based on the Polish legal regulations, and on a simple liming experiment. Total concentrations of metal(loid)s were compared with permissible total concentrations of toxic substances, considered 100% safe, defined by Polish law (Karczewska & Kabała, 2017; Regulation, 2016) (Table S3). They differ among soil groups and subgroups, depending on the land use and on topsoil properties (the content of < 0.02 mm fraction, pH, Corg). A change in soil pH may result in soil transfer to another subgroup with different permissible values. For the deeper soil layers, the permissible values depend mainly on a water permeability, focusing on the protection of groundwater. All these permissible values were established based on the comprehensive studies of bibliography concerning the risk to humans and ecosystems. In the case when the permissible total concentrations are exceeded, Polish law requires more advanced risk analysis to be performed (Karczewska & Kabała, 2017). For this purpose, the effects of changing pH on the actual solubility of elements were examined in a simple laboratory experiment. The problematic samples were mixed with CaCO_3_ (100 g soil + 0.5 g CaCO_3_, which corresponds to ca. 2.8 tons CaO/ha) and incubated for 2 weeks under 80% of water holding capacity). After this time, the concentrations of easily soluble elements, extracted with 1 M NH_4_NO_3_, were determined and assessed based on the literature (ISO 19730; Prüess et al., 1991).

### Statistical analysis

The mean values of actual solubility and PMF for individual elements, expressed as percentage of the total concentrations, were compared between the following groups of samples: a) inter-embankment vs. out-of-embankment sites, b) organic matter-rich top soil layers A vs. deeper horizons with gleyic or stagnic features (Cr, Cg). The data were subject to analysis of variance (ANOVA) followed by Tukey’s test to assess the significance of differences between the means at *P* < 0.05. In order to examine the multivariate relationships between the parameters that characterize soil properties and contributions of soluble fractions of elements, the principal component analysis (PCA) was performed. The raw data on total concentrations of PTEs were log-transformed based on the results of Shapiro–Wilk’s test that revealed the lack of their normal distribution. Log-transformation made the distributions closer to normal. The PCA graphs were generated to illustrate the associations between the variables, as commonly applied (Li et al., 2015; Setia et al., 2021). The number of significant principal components was selected on the basis of the Kaiser criterion with eigenvalue higher than 1. Therefore, only those principal components were retained that indicated the contribution to total variance higher than 10%. The statistical analysis was performed using the StatSoft software Statistica 13.0.

## Results and discussion

### Basic soil properties and total concentrations of metal(loid)s

As assumed, the majority of analyzed samples showed a moderately heavy texture of loams (L) or sandy and silt loams (SL, SiL), while the Cg1 sample, collected from the Out-5 profile, had a texture of clay (C). Soil samples representative of the surface soil layers contained 18–118 g/kg Corg., and the samples from the deeper horizons were much poorer in Corg., and their Corg. content remained below 18 g/kg (Table 1). The soils differed strongly in pH that ranged from 3.4 to 7.4. Both the lowest and the highest pH values were found in the soils of arable fields, in the out-of-embankment zone, while all the inter-embankment samples were either slightly acidic or acidic, and had pH in the range 3.7–5.3. Soil CEC differed widely (in the range 10.4–34.3 cmol^+^ /kg), depending both on the content of Corg and clay fraction in soils.

Total concentrations of Pb, Zn and Cu in topsoil samples from the inter-embankment zone were significantly higher than those in the samples from the out-of-embankment and then in deeper horizons, and significantly exceeded the values considered as the European geochemical background (Table 1). These findings stay in agreement with other studies carried out in the valley of the middle Odra River that proved that the sediments contaminated with heavy metals occur as the layers of various density along the banks of the Odra River, with the larger thickness and width within the former nineteenth- and twentieth-century groin fields (Ciszewski & Grygar, 2016; Ciszewski & Turner, 2009). It is worth noting that in the Int-2 and Int-3 profiles, Pb and Zn concentrations in a top Ah humus horizon, rich in grass roots, were lower than in the underlying Ag or ABw horizons, which can be partly explained by reduced loads of these metals brought recently by the river. The highest concentrations of Pb, Zn and As were present in the ABw sample collected from the Int-3 profile (266, 544, and 45.8 mg/kg, respectively), and the highest Cu concentration, 68.9 mg/kg, was found in the Ah horizon, in the same profile. The most heavy, clay-textured Cr1 sample collected from the Out-5 profile contained the highest concentrations of Mn and Fe, i.e., 2012, and 50,940 mg/kg, respectively.

### Actual solubility of metal(loid)s

The highest amounts of 1 M NH_4_NO_3_- extractable elements were found in the case of Zn and Mn, known as highly mobile (Alloway, 2013; Kabata-Pendias & Szteke, 2015). The maximum values, over 100 mg/kg (Table 2), found in the samples: Int-2,Ag and Int-3,Ah (Zn), and Int-1,Ah (Mn), should be considered extremely high. The concentrations of actually soluble forms of other elements did not exceed 1.0 mg/kg and were the highest in the Int-1,Ah sample.

**Table 2:** Actual solubility of metal(loid)s (determined in 1 M NH_4_NO_3_)

Profile	Horizon	Pb	Zn	Cu	As	Mn	Fe	Pb	Zn	Cu	As	Mn	Fe
				mg/kg					% of total				
Int-1	Ah	0.57	11.5	0.48	0.03	104	0.665	1.6	8.3	2.4	0.21	14.7	0.00
	IIICr	0.04	1.5	0.29	0.03	30.7	< 0.01	0.3	1.5	2.2	0.32	2.5	0.00
Int-2	Ah	< 0.01	28.1	0.29	< 0.01	15.9	< 0.01	0.0	8.7	0.9	0.00	3.9	0.00
	Ag	0.08	107	0.36	< 0.01	24.7	< 0.01	0.1	14.8	0.5	0.00	3.6	0.00
	Bwg	< 0.01	12.7	0.20	< 0.01	19.9	< 0.01	0.0	4.2	0.6	0.00	1.5	0.00
Int-3	Ah	0.09	129	0.45	< 0.01	31.7	< 0.01	0.1	14.1	0.6	0.00	3.8	0.00
	ABw	< 0.01	52.7	0.27	< 0.01	20.6	< 0.01	0.0	4.6	0.5	0.00	1.8	0.00
	IIICg	< 0.01	0.3	0.16	< 0.01	3.0	< 0.01	0.0	0.3	1.7	0.00	0.3	0.00
Out-4	Ap	< 0.01	< 0.01	0.13	< 0.01	9.0	< 0.01	0.0	0.0	2.7	0.00	1.0	0.00
	Cr1	< 0.01	< 0.01	0.20	< 0.01	0.5	< 0.01	0.0	0.0	2.1	0.00	1.3	0.00
Out-5	Ap1	0.16	2.8	0.26	0.03	6.9	< 0.01	0.8	2.5	1.5	0.21	0.8	0.00
	Cg1	< 0.01	0.8	0.26	0.01	10.2	< 0.01	0.0	0.7	1.4	0.09	0.5	0.00
				Average for all samples				0.24	5.0	1.4	0.07	3.0	< 0.001

The shares of actually soluble forms of elements in their total concentrations were also the highest in the case of Zn and Mn, and the average values of those shares decreased in the following order: Zn > Mn > Cu > Pb > As > Fe (Table 2), which confirms the general knowledge on the mobility of elements in the soil environment (Alloway, 2013). It should be noted, however, that the maximum solubility of individual elements, expressed in percentage of total concentrations, was found in various samples, for instance the maximum values of Zn solubility were found in the samples Ag,Int-2, and Ah,Int-3, i.e., the topsoil samples, very rich in Corg., whereas the highest percentages of soluble Mn, Pb and Fe were present in the most strongly acidic Ah,Int-1 sample. The highest share of soluble Cu in its total concentration was found in the Ap,Out-4 sample collected from soil plowed layer, and in the highest share of As - in the IIICr,Int-1 sample with low pH and strongly expressed gleyic properties. This confirms the importance of various factors in governing the actual solubility of various elements.

**Fig. 2:**
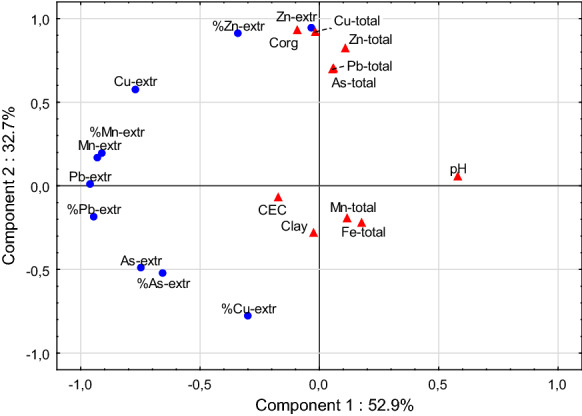
The PCA graph illustrating the relationships between the absolute concentrations (in mg/kg) of easily soluble forms of metal(loid)s, susceptible to extraction with 1 M NH_4_NO_3_ (Me-extr), their percentages in the total concentrations (% Me-extr), the total concentrations in soils (Me-total) and the parameters characterizing soil properties. The graph does not take into account the soluble forms of Fe due to their extremely low share in total concentrations

The results of PCA analysis illustrate these influence of various factors on the actual solubility of elements, expressed in absolute values and percentages (Fig. 2). The pH values were the key factors of a component 1, which had a crucial influence on the solubility of most elements, however, it was of the least importance in the case of Zn and Cu (Fig. 2). The principal component 1 explained 52.9% of total variance. In turn, the solubility of Zn, Cu, and to some extent also As, depended apparently on the factors related to the component 2, that explained 32.7% of variance. The component 2 was associated with the Corg. in soil, and thus with the depth in the profile and the origin of the elements. The percentage of readily soluble Zn was positively correlated with the content of Corg., and it was the highest in surface soil horizons. The opposite relationship was found in the case of Cu, probably due to its strong bonding by humic substances to form insoluble complexes. The solubility of As was also negatively correlated with the component 2 (Fig. 2), which can be explained by the ease of As release from iron oxides under reducing conditions (Gorny et al., 2015; Schulz-Zunkel et al., 2015; Wenzel, 2013), i.e., in the horizons with strong gleyic properties.

### The results of sequential extraction

**Fig. 3:**
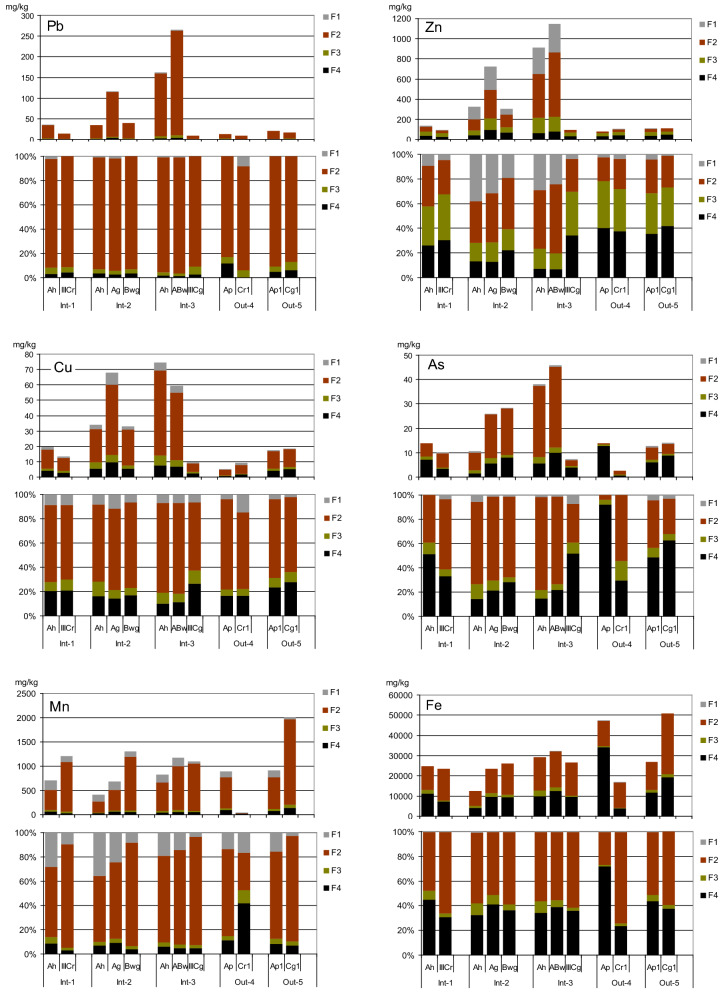
Results of the sequential extraction of Pb, Zn, Cu, As, Mn and Fe in soils. Each pair of graphs illustrates the absolute concentrations and shares of elements in fractions *F*1 (acid-soluble), *F*2 (reducible), *F*3 (oxidizable), and *F*4 (residual)

Three elements, i.e., Zn, Mn and Cu, were present in considerable amounts in the F1 fraction that is susceptible to desorption under acidic conditions (Fig. 3). In the case of Zn, the share of F1 fraction was particularly high in strongly enriched samples, with a clearly anthropogenic origin of this element. Zinc is known to be an easily mobile metal that can be particularly easily released from soil solid phase when the pH drops (Alloway, 2013). The average share of Zn in the *F*1 fraction was 14.4% (Table 3), and the maximum absolute Zn content in this fraction was 129 mg/kg in the Ah horizon of Int-3 profile.

**Table 3:** Average shares of the *F*1:*F*4 fractions, %, and the values of the PMF coefficient, %, for various elements

Element	*F*1	*F*2	*F*3	*F*4	PMF
Pb	1.2	90.5	4.5	3.8	96.2
Zn	14.4	33.5	26.4	25.7	74.3
Cu	7.4	66.2	8.0	18.4	81.6
As	2.4	50.6	7.8	39.2	60.8
Mn	16.0	70.4	4.0	9.6	91.4
Fe	0.3	54.5	5.1	40.1	59.9

The dominant fraction of all elements (except for Zn in soils with its low content) was the reducible *F*2 fraction. Its mean share was especially high, i.e., 90.5%, in the case of Pb (Table 3). It seems a bit surprising, as Pb is believed to have a particularly high affinity to organic matter and to build an organic, oxidizable, fraction (Alloway, 2013, Kabata-Pendias & Szteke, 2015). Similarly high contributions of reducible Pb were, however, reported by numerous authors from the studies carried out with alluvial soils (e.g., Barać et al., 2016; Kanianska et al., 2022; Różański, 2013). Also Sutherland and Tack (2002) reported *F*2 as a dominant Pb fraction in five certified reference soils. The predominance of reducible Pb species in soils should be subjected in the future to a more in-depth analysis and interpretation.

The only element in our research that showed a relatively high contribution of the oxidizable fraction *F*3 was Zn, with its average share amounting to 26.4%. This result is also quite surprising, as the affinity of Zn to organic matter is known to be much lower than that of Cu or Pb. One would expect rather that the oxidizable *F*3 fraction would be the main Cu species. In our research, however, the share of *F*3 fraction of Cu was relatively low, in the range 5.2–12.5%, with the mean value of only 8.0%.

As mentioned earlier, the patterns of Zn and Cu fractionation reported in the literature are highly differentiated. Li et al., (2015) showed that over 90% of Zn and Cu in soil profiles in the Yellow River delta were present in the residual fraction (*F*4). Similarly to our results, Różański (2013) and Frentiu et al., (2009) reported the predominance of Cu in *F*2 over *F*3 in alluvial soils. Similar relationships were also found by Sutherland and Tack (2002) in three out of five certified reference soils, whereas in the remaining two soils, the relationships were opposite. Shaheen and Rinklebe (2014), based on the sequential extraction according to Zeien and Brümmer, indicated that the dominant Cu fraction in various types of German and Egyptian soils was organically bound, but in the light of BCR extraction it was not so obvious (Rinklebe & Shaheen, 2017). Numerous authors who applied the BCR procedure to examine the forms of Pb, Cu, and Zn in various types of soils reported the dominance of the organic *F*3 fraction of these metals over the reducible one *F*2 (Boim et al., 2021; Gholami & Rahimi, 2021; Karczewska et al., 2005; Kubova et al., 2008; Liu et al., 2022; Memoli et al., 2018; Sungur et al., 2014). This was true in most soils where the origin and accumulation of these elements were not related to the process of sedimentation in water, and the paper by Barać et al., (2016) was the exceptional one that confirmed a higher share of *F*3 than *F*2 in alluvial soils.

On the contrary to Pb, Zn and Cu, the results of BCR analysis of As, Mn and Fe, and in particular the high shares of *F*2 fraction (Table 3), stay in agreement with the basic knowledge and various bibliographic reports. The susceptibility of Mn and Fe to reductive dissolution, and the fact that Fe (hydro)oxides are the main sinks of As in soils and sediments (Alloway, 2013; Lewińska et al., 2019; Wenzel, 2013; Xu et al., 2017) explain well the predominance of *F*2 fraction of these elements. Significant amounts of Fe and As were in this study present also in the *F*4, residual, fraction, unlike Mn, whose share in the *F*4 fraction was very low.

In summary, it is worth emphasizing that the BCR procedure is fully operationally defined, and therefore its results do not reflect the real geochemical forms of elements. It is disputable if the reducible *F*2 fraction corresponds to the species occluded in Fe and Mn oxides, and the oxidizable *F*3 fraction to organically bound forms, as commonly interpreted. However, the large share of reducible fraction *F*2 of all elements in our soils is striking and worth attention It stays with agreement with the review made by Du Laing et al., (2009), concerning the fate of trace elements in alluvial soils. They stressed that the processes of co-precipitation with Mn and Fe (hydro)oxides, formed in the hydrolysis reactions, and deposited or transported as river sediments, are of particular importance, which makes these hydroxides, susceptible to reduction, an important sink of various metal(loid)s accumulated in alluvial soils.

Based on the results of the sequential extraction, the PMF was calculated (Table 3). The average values of PMF for various elements were in the order: Pb > Mn > Cu > Zn > Fe > As, which was partly unexpected. The maximum value of PMF (96.2%) was found for Pb, which raises no doubts and is consistent with the research of many authors (Barać et al., 2016; Kanianska et al., 2022; Rinklebe & Shaheen, 2014; Różański, 2013). Such a high mobility of Pb can be explained by its mainly anthropogenic origin. The high potential mobility of Mn, second in line, is also commonly known and was very often reported. An unexpected result, however, was the higher average PMF value for Cu compared with Zn, which is considered a highly mobile element originated in the Odra river basin mainly from the sources related to Zn and Pb mining and processing (Ciszewski & Grygar, 2016). The lowest values of PMF (ca. 60% on average) were found for As and Fe, i.e., the elements known as poorly mobile.

**Fig. 4:**
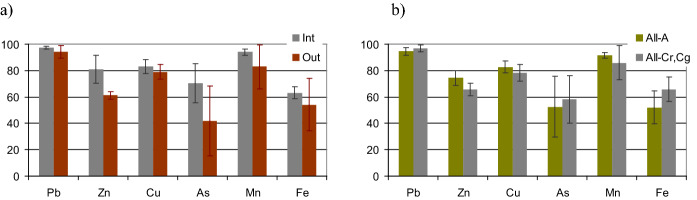
Mean values of potential mobility factor (PMF), %, for various elements. The comparison between: a) inter-embankment (Int) vs. out-of-embankment (Out) zones, b) surface soil layers enriched in humus (A) vs. deeper horizons, with redoximorphic, i.e., gleyic or stagnic features (Cr, Cg). Error bars indicate confidence intervals (*P* > 0.95)

The PMF values were compared between the groups of samples representing the inter- and out-of-embankment zones, and between the samples from the surface and deeper soil horizons (Fig. 4). The only statistically significant difference (p > 95%) was found for Zn that indicated a higher PMF in the inter-embankment compared with the out-of-embankments zone.

### Assessed environmental risk

The first approach to assess the risk involved calculation of geochemical contamination indices EF, RI and Igeo. The EF index fell into the class of soil significant enrichment in Pb and Zn in humus horizons of two inter-embankment profiles Int-2 (Ah and Ag) and Int-3 (Ah and ABw). Additionally, the Ah horizon in the Int-3 profile indicated significant enrichment in Cu (Table 4). Lower, i.e., moderate enrichment in four elements, was found in the Bwg sample in this profile, while in all the remaining samples the EF index was below 2, i.e., the samples were not enriched. None of the samples showed very high or extreme enrichment in the elements under study (Table S2). The assessment based on the Igeo was basically similar, with this index showing the highest enrichment in Pb in the ABw,Int-3 sample, classified as heavily contaminated. This was the only sample, for which the overall ecological risk was assessed as moderate (RI > 150, Table 4), mainly because of high individual risk index for Pb (Er_i_ > 80, Table S4).

**Table 4:** Geochemical indices of soil contamination with PTEs and classes of associated risk

Profile	Horizon		EF^a)^					Igeo^b)^				RI	
		Pb	Zn	Cu	As	Mn	Pb	Zn	Cu	As	Mn	Value	Risk
Int-1	Ah	3.1	1.6	1.7	1.5	1.1	1.3^I^	0.4	0.5	0.3	− 0.19	33	Low
	IIICr	1.3	1.1	1.2	1.2	1.9	− 0.04	− 0.2	− 0.1	− 0.1	0.57	20	Low
Int-2	Ah	5.3^++^	6.8^++^	4.9^+^	2.1^+^	1.1	2.3^II^	2.6^II^	2.1^II^	0.9^I^	− 0.04	71	Low
	Ag	10.5^++^	8.7^++^	5.0^+^	3.0^+^	1.1	3.3^II^	3.1^II^	2.3^II^	1.5^I^	0.02	111	Low
	Bwg	3.3^+^	3.3^+^	2.3^+^	2.9^+^	1.9	1.6^I^	1.6^I^	1.0^I^	1.4^I^	0.78	55	Low
Int-3	Ah	12.1^++^	9.0^++^	5.4^++^	3.6^+^	1.1	3.5^II^	3.0^II^	2.3^II^	1.7^I^	− 0.02	122	Low
	ABw	17.9^++^	10.2^++^	3.9^+^	3.9^+^	1.4	4.2^III^	3.4^II^	2.0^I^	2.0^I^	0.52^I^	166	Moderate
	IIICg	0.7	1.0	0.8	0.7	1.5	− 0.5	− 0.1	− 0.5	− 0.6	0.54	15	Low
Out-4	Ap	0.5	0.5	0.2	0.7	0.6	0.6^I^	0.5	− 0.5	1.2^I^	0.95^I^	35	Low
	Cr1	1.2	1.7	1.2	0.4	0.1	− 0.2	0.3	− 0.2	− 1.8	− 3.96	13	Low
Out-5	Ap1	1.6	1.2	1.4	1.3	1.3	0.5	0.01	0.3	0.1	0.14	25	Low
	Cg1	0.7	0.6	0.8	0.8	1.5	0.2	− 0.1	0.2	0.2	1.21 ^I^	24	Low

^a)^Categories of EF (for details see: Supplementary Materials, Table S2)

(no superscript): no enrichment (EF < 2)

^+^moderate enrichment (EF: 2–5)

^++^significant enrichment (EF: 5–20)

^b)^Categories of Igeo (for details see: Supplementary Materials, Table S2): 0: Igeo < 0 and 0–0.5, unpolluted soils; I: Igeo 0.5–2.0, slight pollution; II: Igeo 2.0–3.5, moderate pollution; III: Igeo 3.5–4.5, heavy pollution; IV: Igeo > 4.5, extreme pollution

The second approach, based on Polish law (Regulation, 2016), produced the picture that partly agrees with that described above. Permissible soil concentrations of 11 metals (Ba, Cd, Co, Cr, Cu, Hg, Mo, Ni, Pb, Sn, and Zn), and As—a metalloid, have been set separately for various categories of land usage. The environmental risk assessed for pastures and meadows and arable lands relates mainly to the possible uptake of PTEs by food or forage plants, to a direct impact on soil biota and to leaching to surface or groundwater. Therefore, the permissible concentrations of PTEs in the top soil layer (0–25 cm) differ depending on soil texture, organic matter content and pH, and those in the deeper soil layers (> 25 cm) are related to water permeability (Table S3). In three of the samples, representing the profiles Int-2 and Int-3, situated in the inter-embankment zone, the concentrations of Zn and As exceeded the values considered 100% safe. Permissible Zn and As concentrations in topsoil (0–25 cm) used for agricultural purposes and containing more that, 20% of < 0.02 mm fraction, have been set at 500 and, 20 mg/kg at pH < 5.5 and 1000 and 50 mg/kg at pH > 5.5, respectively (Table S3). Permissible concentrations of Zn and As in subsoil (> 25 cm), other than sand and gravel, are 500 and 50 mg/kg, respectively (Table S3). The concentrations of Zn and As in two topsoil samples (Ag,Int-2 and Ah,Int-3) exceeded the permissible levels, mainly because of low soil pH (< 5.5), which was crucial for the placement of soils in the subgroups (Table S4). It is absolutely necessary, therefore, to lime acidic soils in the inter-embankment zone in order to neutralize the acidic reaction. After carrying out this treatment and raising the pH to a value > 5.5, these soils will meet the requirements of Polish law and will be considered as not posing a risk associated with their agricultural usage. This fact was confirmed by a laboratory liming experiment, in which the concentrations of easily soluble Zn, extracted with 1 M NH_4_NO_3_, decreased from 107 and 129 mg/kg, respectively, to below 2.0 mg/kg, and the As concentration in the extract remained below the quantification limit (< 0.05 mg/kg).

The only problematic sample was the ABw sample from the Int-3 profile (30–55 cm), with the very high concentration of Zn: 1145 mg/kg, exceeding the permissible value (that in the case of subsoil does not depend on pH). In such case, Polish law requires a detailed risk analysis to be performed, taking into account the exposure routes for humans, animals and ecosystems. Such an analysis would go beyond the scope of this article. However, the liming experiment proved that the concentration of actually soluble Zn forms in this sample dropped from 52.7 mg/kg (at pH 5.3) to 1.8 mg/kg at pH 7.2. This makes it possible to conclude that soil liming will allow to effectively reduce the risk of Zn mobilization to water. Lack of stagnic features in the ABw horizon, together with the fact that Zn occurred in the ABw sample mainly in the reducible forms (*F*2 fraction), makes us conclude that after liming, the risk of Zn release from the ABw horizon will be negligible. It is also worth noting that this particular sample, ABw,Int-3, showed also an elevated, i.e., moderate, risk in the light of the RI-based assessment (Table 4), however the decisive element was in that case Pb rather than Zn, because of a toxicity factor assigned to Pb (Tr = 5), five times higher than that for Zn (Table S2, S5).

## Summary and conclusions

This analysis indicates that single extraction and fractionation should play important role in assessing the risk caused by PTEs present in soils, especially if geochemical indices of contamination are elevated. The analysis of operationally defined forms of metal(loid)s in the alluvial soils of the Odra River, performed by the BCR sequential extraction, showed a very high share of reducible fraction (*F*2) of all elements, which suggests that the key process leading to their accumulation in alluvial soils was probably the co-precipitation and occlusion in manganese and iron (hydroxy)oxides, formed as river sediments, that were further transported and deposited in the floodplains. Moreover, the predominance of *F*2 fraction of elements indicates that they can be potentially released from soils under reducing conditions, which refers particularly to the horizons bearing gleying or stagnic features. Two elements, Zn and Mn, had relatively high shares of acid-soluble *F*1 fraction, particularly in the topsoils of the inter-embankment zone. Actual solubility of Zn and Mn, determined in extraction with 1 M NH_4_NO_3_, was also assessed in those cases as high, mainly due to low soil pH (3.7–5.3). It was proved, however, that soil liming will significantly reduce this solubility thus reducing also the environmental risk.

Although Pb and Zn concentrations in some samples of soil humus horizons in the inter-embankment zone were classified, based on EF and Igeo indices, as elevated, and the potential ecological risk determined based on total concentrations was in one sample assessed as moderate, the results of fractionation indicated that the real risk can be reduced in these soils to acceptable level, provided that effective liming is applied. The lack of gleyic or stagnic features in the humus horizons of the most enriched soils indicates that reducing conditions in fact do not occur there, and therefore the risk that the reducible forms of PTEs, in particular Pb and As, will be released, is negligible.

### Supplementary Information

Below is the link to the electronic supplementary material.

Supplementary file1 (DOC 120 KB)

## Data Availability

The data that support the findings of this study are available on request from Dr. Dorota Kawałko.
