# Supplementary material for: Environmental risk associated with accumulation of toxic metalloids in soils of the Odra River floodplain—case study of the assessment based on total concentrations, fractionation and geochemical indices
Source: Environ Geochem Health. 2023 Feb 23;45(7):4461–76. doi: 10.1007/s10653-023-01502-1 (PMC10310573; doi:10.1007/s10653-023-01502-1)
Supplement: Supplementary file 1 — Supplementary file1 (DOC 120 KB) [file 10653_2023_1502_MOESM1_ESM.doc]

**Environmental risk associated with accumulation of toxic metalloids in soils of the Odra river floodplain – case study of the assessment based on total concentrations, fractionation and geochemical indices**

Dorota Kawałko ^1^, Anna Karczewska ^1^*, Karolina Lewińska ^2^

^1^ Wrocław University of Environmental and Life Sciences, Institute of Soil Science, Plant Nutrition and Environmental Protection, ul. Grunwaldzka 53, 50-357 Wrocław, Poland;

^2^ Adam Mickiewicz University in Poznań, Department of Soil Science and Remote Sensing of Soils, ul. Krygowskiego 10, 61-680 Poznań, Poland;

* Corresponding autor: [anna.karczewska@upwr.edu.pl](mailto:anna.karczewska@upwr.edu.pl)

**SUPPLEMENTARY MATERIALS**

**Table S1.** Detailed procedure of the modified BCR sequential extraction procedure with an *aqua regia* digestion (Rauret el al., 1999)

| Fraction | Operational definition | Chemical reagents and conditions |
| --- | --- | --- |
| F1 | Acid extractable | 1 g of soil sample, 40 ml 0.11 M acetic acid, shake for 16 h at 22 ± 5ºC. Separate extract from the solid residue by centrifugation at 3000 g for 20 min |
| F2 | Reducible | To step 1 residue, add 40 ml 0.5 M NH_2_OH·HCl from a 1 l solution containing 25 ml 2 M HNO_3_ (pH ≈ 1.5), shake for 16 h at 22 ± 5ºC. Centrifuge extract as per step 1 |
| F3 | Oxidizable | To step 2 residue, add 10 ml H_2_O_2_ (pH 2–3), 1 h at room temperature, heat to 85 ± 2ºC for 1 h; add a further 10 ml H_2_O_2_ and heat to 85 ± 2ºC for 1 h; add 50 ml; 1 M NH_4_OAc (pH 2) and shake for 16 h at 22 ± 5ºC. Centrifuge extract as per step 1 |
| F4 | Residual | To step 3 residue, add max. 3 ml distilled H_2_O, 7.5 ml 6 M HCl, and 2.5 ml 14 M HNO_3_; leave overnight at 20ºC, boil under reflux for 2 h, cool and filter |

**Table S2. Geochemical indices of soil contamination - formulas and classes for assessment**

| Index name | Formula | Classes | Source | Comments* |
| --- | --- | --- | --- | --- |
| Enrichment factor  EF | EF = [Cs/CsRef]/[GB/GBRef] where:  Cs - concentration of element in soil;  CsFe – concentration of reference element in soil;  GB - geochemical background of element GBRef - geochemical background of the reference element. | EF < 2: no enrichment  EF: 2-5: moderate enrichment  EF: 5-20: significant enrichment  EF: 20-40: very high enrichment  EF > 40: extremely high enrichment | Barbieri (2016) | A reference element is an element particularly stable in soil. In this paper, Fe was used as a reference element as suggested by many authors.  An exact formula for calculation of EF was therefore: EF = [Cs/Cs_Fe_]/[GB/GB_Fe_]. |
| Index of geoaccumu-lation  Igeo | Igeo =  log_2_[Cs /(1.5⋅GB)]  where:  Cs: concentration of element in soil;  GB: geochemical background.  1.5 – constant that allows to take into account a natural variability in the environment | Original classes of “contamination”  Igeo < 1: uncontaminated  Igeo 0-1: uncontaminated to moderately contaminated  Igeo 1-2: moderately c.  Igeo 2-3: moderately to heavily c.  Igeo 3-4: heavily c.  Igeo 4-5: heavily to extremely c.  Igeo > 5: extremely c. | Müller (1981) | The factor 1.5 is redundant in the case when a local background is used for calculations of Igeo.  Precisely defined classes of soil contamination:  Igeo < 0.5: uncontaminated  Igeo 0.5-2.0: slightly c.  Igeo 2.0-3.5: moderately c.  Igeo 3.5-4.5: heavily c.  Igeo >4.5: extremely c. |
| Potential ecological risk index  RI | RI = ΣER_i_,  where  ER_i_: individual risk of particular (i) element  ER_i_ = Tr_i_ x Cf_i_  Cf_i_: pollution coefficient  Cf_i_ = Cs / GB  Tr_i_ : toxicity coefficient of element: Pb: 5, Zn: 1, Cu: 5, As:10 | RI < 150: low ecological risk  RI 150-300: moderate risk  RI 300-600: considerable risk  RI > 600: high risk or danger  For particular elements:  ER_i_ < 40 : low ecological risk  ER_i_ 40-80: moderate risk  ER_i_ 80-160; considerable risk  ER_i_ 160-320: high risk  ER_i_ > 320 dangerous | Hakanson (1980) | Mn and Fe are not considered as potentially toxic elements in this approach to the assessment of ecological risk. |

* According to originally proposed definitions of indices, the values of geochemical background GB are the average concentration in the Earth’s crust. However, as suggested by various authors, for the assessment of soil contamination, they should be replaced by local values of background concentrations of particular elements. Therefore, for calculations of all the indices in this paper, the local values of background concentrations in the valley of the Odra river, dependent on soil textures, presented in another paper by Kawałko and Karczewska (submitted), were used.

**Table S3.** Permissible concentrations of PTEs, considered by Polish law unconditionally safe, in the group of soils used for agricultural purposes, i.e. group II (Regulation, 2016).

| Element | Depth 0-0.25 m | | | Depth > 0.25 m | |
| --- | --- | --- | --- | --- | --- |
|  | Soil subgroups ^a)^ | | | Water permeability ^b)^ | |
|  | II-1 | II-2 | II-3 | High | Low |
| As | 10 | 20 | 50 | 20 | 50 |
| Cu | 100 | 150 | 300 | 150 | 300 |
| Pb | 100 | 250 | 500 | 100 | 300 |
| Zn | 300 | 500 | 1000 | 300 | 500 |

^a)^ Soil subgroups dependent on the content of organic matter, texture (the content of fraction <0.02 mm), and pH:

| Soil organic matter, % | Content of fraction  < 0.02 mm, % | pH (1M KCl) | | | |
| --- | --- | --- | --- | --- | --- |
|  |  | < 4.5 | 4.5-5.5 | 5.5-6.5 | >6.5 |
| <6 | <10 | II-1 | II-1 | II-1 | II-1 |
|  | 10-20 | II-1 | II-1 | II-1 | II-2 |
|  | >20 | II-2 | II-2 | II-3 | II-3 |
| 6-10 | irrelevant | II-2 | II-2 | II-2 | II-2 |
| > 10 |  | II-3 | II-3 | II-3 | II-3 |

^b)^ The permissible concentrations of PTEs in subsurface soil layers (deeper than 25 cm) depend on water permeability. The values ≥10^-7^ m/s are considered high, the values < 10^-7^ m/s – low.

**Table S4.** Detailed data on the samples in which the permissible Zn and As concentrations (considered by Polish law unconditionally safe) were exceeded.

| Profile  Land use | Hori-zon | Depth  cm | Textural class | Clay  % | F02^a)^  % | pH | Sub-group ^b)^ | Zn  mg/kg | Permissible Zn mg/kg | | As  mg/kg | Permissible As mg/kg | |
| --- | --- | --- | --- | --- | --- | --- | --- | --- | --- | --- | --- | --- | --- |
|  |  |  |  |  |  |  |  |  | pH<5.5 | pH>5.5 |  | pH<5.5 | pH>5.5 |
| Int-2 Pasture | Ag | 16-30 | SiL | 12 | 40 | 5.0 | II-2 | 724 | 500 | 1000 | 26.1 | 20 | 50 |
| Int-3 | Ah | 0-15 | SiL | 25 | 65 | 4.6 | II-2 | 914 | 500 | 1000 | 38.2 | 20 | 50 |
| Meadow | ABw | 30-55 | SiL | 20 | 63 | 5.3 | II-Low | 1145 | 500 | 500 | 45.8 | 50 | 50 |

^a)^ F02 – fraction <0.02 mm

^b)^ The group and subgroups according to Polish law (see: Table S3)

Gray background indicates the cases where the permissible concentrations of Zn and As are exceeded

**Table S5.** Detailed data on the values of ERi (the indices of potential risk for individual elements), calculated according to Hakanson (1980)

| Profile | Horizon | ER_i_ | | | | Assessment of ecological risk,  based on ER_i_  values |
| --- | --- | --- | --- | --- | --- | --- |
|  |  | Pb | Zn | Cu | As |
| Int-1 | Ah | 13 | 1 | 7 | 12 | Low |
|  | IIICr | 5 | 1 | 5 | 9 | Low |
| Int-2 | Ah | 24 | 6 | 22 | 19 | Low |
|  | Ag | 50 | 8 | 24 | 28 | Moderate for Pb, low else |
|  | Bwg | 15 | 3 | 10 | 27 | Low |
| Int-3 | Ah | 56 | 8 | 25 | 33 | Moderate for Pb, low else |
|  | ABw | 94 | 11 | 20 | 41 | Considerable for Pb, moderate for As, low else |
|  | IIICg | 3 | 1 | 4 | 7 | Low |
| Out-4 | Ap | 8 | 1 | 3 | 22 | Low |
|  | Cr1 | 4 | 1 | 4 | 3 | Low |
| Out-5 | Ap1 | 7 | 1 | 6 | 11 | Low |
|  | Cg1 | 6 | 1 | 6 | 12 | Low |
